# Supplementary material for: Gene Expression in Parthenogenic Maize Proembryos
Source: Plants (Basel). 2021 May 12;10(5):964. doi: 10.3390/plants10050964 (PMC8151209; doi:10.3390/plants10050964)
Supplement: Supplementary file 1 [file plants-10-00964-s001.zip › plants-1204104-supplementary.pdf]

Supplementary Materials

Table S1. **List of the metabolites analysed in this study**. Metabolites in bold were below the limit of detection under our experimental conditions.

Phytohormone group	***Abbreviations***	***Name***
Auxins	IAA	indole-3-acetic acid
	oxIAA	2-oxindole-3-acetic acid
	IAAsp	indole-3-acetyl-L-aspartic acid
	IAGlu	indole-3-acetyl-glutamic acid
Isoprenoid cytokinins	*t*Z	*trans*-zeatin
	*t*ZR	*trans*-zeatin riboside
	*t*ZRMP	*trans*-zeatin riboside-5'-monophosphate
	***t*Z9G**	***trans*-zeatin-9-glucoside**
	***t*Z7G**	***trans*-zeatin-7-glucoside**
	*t*ZOG	*trans*-zeatin-*O*-glucoside
	*t*ZROG	*trans*-zeatin riboside-*O*-glucoside
	**DHZ**	**dihydrozeatin**
	**DHZR**	**dihydrozeatin riboside**
	**DHZRMP**	**dihydrozeatin riboside-5'-monophosphate**
	**DHZ9G**	**dihydrozeatin-9-glucoside**
	**DHZ7G**	**dihydrozeatin-7-glucoside**
	DHZOG	dihydrozeatin-*O*-glucoside
	**DHZROG**	**dihydrozeatin riboside-*O*-glucoside**
	*c*Z	*cis*-zeatin
	***c*ZR**	***cis*-zeatin riboside**
	***c*ZRMP**	***cis*-zeatin riboside-5'-monophosphate**
	***c*Z7G**	***cis*-zeatin-7-glucoside**
	***c*Z9G**	***cis*-zeatin-9-glucoside**
	*c*ZOG	*cis*-zeatin-*O*-glucoside
	***c*ZROG**	***cis*-zeatin riboside-*O*-glucoside**
	iP	isopentenyladenine
	iPR	isopentenyladenenosine
	iPRMP	isopentenyladenosine-5'-monophosphate
	**iP9G**	**isopentenyladenine-9-glucoside**
	iP7G	isopentenyladenine-7-glucoside

Table S2. Levels of endogenous auxin metabolites (pmol g^{-1} FW) in the hypocotyl and developmental root sections of cacao seedlings harvested at 4, 7 and 10 DAI. A, B, and C indicate the biological replicates. Mz = meristematic zone; Ez = elongation zone; Dz = differentiation zone bearing protrusions of lateral roots; mDz = mature differentiation zone; H = hypocotyl segment. A dash (-) indicates the absence of values as the mDz segment was not formed at day 4. FW = fresh weight.

		Content of IAA (pmol g^{-1} FW) in different tissues					***Content of IAAsp (pmol g^{-1} FW) in different tissues***				
DAI		**Mz**	**Ez**	**Dz**	**mDz**	**H**	**Mz**	**Ez**	**Dz**	**mDz**	**H**
4	A	310.96	378.47	116.81	-	38.91	1505,88	1208,06	1935,43	-	764,28
	B	339.33	134.72	106.88	-	79.30	1289,25	646,05	570,05	-	285,47
	C	159.92	88.24	66.61	-	25.34	1202,31	700,56	1373,12	-	181,71
	**Mean values**	**270,07**	**200,48**	**96,77**	**-**	**47,85**	**1332,48**	**851,56**	**1292,86**	**-**	**410,48**
7	A	224.55	75.93	67.963	52.317	34.261	1112,34	1405,85	1153,35	562,88	783,08
	B	204.19	152.43	117.506	72.051	100.062	357,33	300,05	449,54	732,35	36,06
	C	206.60	89.45	99.831	34.703	45.686	702,107	859,52	903,77	769,38	514,56
	**Mean values**	**211,78**	**105,94**	**95,09**	**53,02**	**60,00**	**723,93**	**855,14**	**835,55**	**688,20**	**444,57**
10	A	18.39	36.46	90.07	19.45	8.77	1309,87	2616,56	614,59	2386,31	703,50
	B	20.87	29.37	77.84	37.93	29.95	2249,92	1053,08	1515,12	102,22	145,18
	C	19.38	14.69	32.77	21.40	18.32	2030,387	1255,11	1195,28	337,36	187,63
	**Mean values**	**19,54**	**26,84**	**66,89**	**26,26**	**19,01**	**1863,39**	**1641,59**	**1108,33**	**941,96**	**345,44**
		Content of free IAGlu (pmol g^{-1} FW) in different tissues					***Content of oxIAA (pmol g^{-1} FW) in different tissues***				
4	A	10.20	14.17	32.18	-	5.39	13.42	16.01	1.86	-	1.14
	B	6.60	4.05	14.17	-	1.56	8.20	6.74	5.62	-	4.34
	C	7.17	5.49	34.69	-	1.46	6.85	12.39	10.80	-	8.48
	**Mean values**	**7.99**	**7.90**	**27.01**	**-**	**2.80**	**9.49**	**11.71**	**6.09**	**-**	**4.65**
7	A	29.84	37.76	27.76	42.57	6.96	3.00	1.78	3.18	1.70	1.22
	B	2.79	6.24	6.22	15.45	6.31	9.38	8.79	7.59	3.22	7.46
	C	7.24	12.17	13.09	14.77	5.36	8.74	4.63	2.95	3.22	1.66
	**Mean values**	**13.29**	**18.72**	**15.69**	**24.26**	**6.21**	**7.04**	**5.07**	**4.58**	**2.71**	**3.45**
10	A	15.62	38.46	18.07	48.97	7.47	16.75	23.05	54.83	16.74	8.69
	B	48.67	20.59	39.22	12.63	9.10	21.23	21.34	53.60	21.05	12.21
	C	72.87	51.80	25.46	13.77	7.72	20.20	19.94	29.13	24.65	12.98
	**Mean values**	**45.72**	**36.95**	**27.58**	**25.12**	**8.10**	**19.39**	**21.44**	**45.85**	**20.81**	**11.30**

Table S3. Levels of endogenous cytokinins (pmol/g fresh weight) in the hypocotyl and developmental root sections of cacao seedlings harvested at 4, 7 and 10 DAI. A, B, and C indicate the biological replicates. “<LOD” indicates values below the limit of detection of UHPLC-MS/MS method used. Mz = meristematic zone; Ez = elongation zone; Dz = differentiation zone bearing protrusions of lateral roots; mDz = mature differentiation zone; H = hypocotyl segment. A dash (-) indicates the absence of values as the mDz segment was not formed at day 4. FW = fresh weight.

DAI	*Plant tissue*		**iP**	**iPR**	**iP7G**	**iPRMP**	***t*Z**	***t*ZR**	***t*ZOG**	***t*ZROG**	***t*ZR5'MP**	**DHZOG**	***c*Z**	***c*ZOG**
**4**	**Mz**	A	1.652	4.351	0.930	1.612	0.044	0.206	**<LOD**	0.040	0.708	0.006	0.011	**<LOD**
		B	1.680	4.599	0.230	2.062	0.091	0.641	0.071	0.065	1.796	**<LOD**	0.011	**<LOD**
		C	0.735	2.383	0.089	0.807	0.082	0.371	0.150	0.051	1.230	0.002	**<LOD**	**<LOD**
	**Mean values**		**1.355**	**3.778**	**0.416**	**1.493**	**0.073**	**0.406**	**0.110**	**0.052**	**1.245**	**0.004**	**0.011**	<LOD
	**Ez**	A	1.059	2.411	0.377	1.429	0.288	1.688	0.257	0.077	5.170	0.008	0.015	**<LOD**
		**B**	0.332	2.887	0.223	0.636	0.109	1.475	0.078	0.019	1.867	0.003	0.012	**<LOD**
		**C**	0.623	2.321	0.230	0.423	0.145	0.913	0.421	0.065	1.368	0.004	**<LOD**	**<LOD**
	**Mean values**		**0.671**	**2.539**	**0.277**	**0.830**	**0.181**	**1.359**	**0.252**	**0.054**	**2.802**	**0.005**	**0.014**	<LOD
	**Dz**	A	**<LOD**	**<LOD**	0.510	0.297	0.399	1.212	0.720	0.086	1.721	0.097	0.014	**<LOD**
		B	0.505	1.651	0.088	0.604	0.296	1.392	0.438	0.059	2.378	0.012	0.005	**<LOD**
		C	0.707	2.964	0.143	0.915	0.257	0.825	0.435	0.066	1.945	0.024	**<LOD**	**<LOD**
	**Mean values**		**0.606**	**2.308**	**0.247**	**0.605**	**0.318**	**1.143**	**0.531**	**0.070**	**2.014**	**0.045**	**0.010**	<LOD
	**H**	A	0.322	0.690	0.155	0.196	0.809	1.471	0.881	0.082	4.094	0.056	**<LOD**	**<LOD**
		B	0.299	1.729	0.133	0.416	0.612	2.055	0.357	0.059	4.731	0.009	**<LOD**	**<LOD**
		C	0.141	1.116	***<LOD***	0.162	0.561	1.470	0.646	0.072	3.202	0.022	**<LOD**	**<LOD**
	**Mean values**		**0.254**	**1.178**	**0.144**	**0.258**	**0.661**	**1.665**	**0.628**	**0.071**	**4.009**	**0.029**	<LOD	<LOD
**7**	**Mz**	A	0.454	1.817	0.097	0.548	0.116	1.361	0.296	0.033	2.364	0.005	**<LOD**	**<LOD**
		B	0.905	1.954	0.149	0.627	0.244	0.726	0.199	0.029	1.856	0.007	**<LOD**	**<LOD**
		C	0.915	2.838	0.063	0.406	0.226	0.850	0.363	0.026	2.279	0.006	0.002	**<LOD**
	**Mean values**		**0.758**	**2.203**	**0.103**	**0.527**	**0.196**	**0.979**	**0.286**	**0.029**	**2.167**	**0.006**	**0.002**	<LOD
	**Ez**	A	0.265	1.237	0.102	0.143	0.183	1.562	1.707	0.067	1.878	0.015	**<LOD**	**<LOD**
		**B**	0.759	1.567	1.236	0.331	0.295	1.802	1.286	0.066	2.801	0.010	0.086	**<LOD**
		**C**	0.724	2.721	0.095	0.444	0.357	2.812	0.943	0.049	2.938	0.012	0.047	**<LOD**
	**Mean values**		**0.583**	**1.841**	**0.478**	**0.306**	**0.278**	**2.058**	**1.312**	**0.061**	**2.539**	**0.013**	**0.067**	<LOD

Table S3 Continued

DAI	*Plant tissue*		iP	iPR	iP7G	iPRMP	*t*Z	*t*ZR	*t*ZOG	*t*ZROG	*t*ZR5'MP	DHZOG	*c*Z	*c*ZOG
**7**	**Dz**	A	0.453	0.927	0.075	0.169	0.114	1.347	0.988	0.076	1.027	0.032	0.018	**<LOD**
		B	0.492	1.462	0.067	0.259	0.371	1.819	0.640	0.071	1.216	0.019	**<LOD**	**<LOD**
		C	0.002	**<LOD**	0.104	0.212	0.223	0.978	0.392	0.037	1.199	0.009	0.001	**<LOD**
	**Mean values**		**0.316**	**1.195**	**0.082**	**0.213**	**0.236**	**1.381**	**0.674**	**0.061**	**1.148**	**0.020**	**0.009**	**<LOD**
	**mDz**	A	0.390	1.811	0.281	0.208	0.122	3.229	1.597	0.100	1.657	0.035	0.008	**<LOD**
		B	1.057	2.881	0.312	0.416	0.328	2.969	1.224	0.100	1.908	0.028	**<LOD**	**<LOD**
		C	0.995	2.212	0.417	0.344	0.374	2.529	1.458	0.054	2.468	0.030	**<LOD**	**<LOD**
	**Mean values**		**0.814**	**2.301**	**0.337**	**0.323**	**0.275**	**2.909**	**1.426**	**0.084**	**2.011**	**0.031**	**0.008**	**<LOD**
	**H**	A	0.356	0.911	0.060	0.335	0.312	0.892	0.489	0.060	2.193	0.015	**<LOD**	**<LOD**
		B	0.250	0.475	0.011	0.307	0.725	2.567	0.955	0.069	4.765	0.016	0.188	**<LOD**
		C	0.226	1.167	0.312	0.307	0.604	1.677	0.482	0.058	4.223	0.007	0.111	**<LOD**
	**Mean values**		**0.277**	**0.851**	**0.128**	**0.317**	**0.547**	**1.712**	**0.642**	**0.062**	**3.727**	**0.013**	**0.150**	**<LOD**
**10**	**Mz**	A	**<LOD**	3.746	***<LOD***	0.047	0.039	0.272	0.462	**<LOD**	**<LOD**	0.011	0.034	0.0000
		B	0.423	1.419	0.216	0.193	0.068	0.282	3.064	0.206	0.889	0.025	0.043	0.0044
		C	0.192	1.039	0.322	0.220	0.058	0.294	1.094	0.074	1.266	0.008	**<LOD**	0.1164
	**Mean values**		**0.308**	**2.068**	**0.269**	**0.127**	**0.055**	**0.283**	**1.540**	**0.140**	**1.077**	**0.015**	**0.038**	**0.040**
	**Ez**	A	0.497	1.864	0.232	0.160	0.232	1.756	3.871	0.280	5.862	0.043	0.022	0.0000
		B	0.389	0.645	0.174	0.047	0.044	0.252	1.419	0.080	0.568	0.009	0.051	0.0170
		C	0.160	1.077	0.477	0.174	0.121	0.588	2.500	0.120	1.864	0.019	0.011	0.6060
	**Mean values**		**0.349**	**1.195**	**0.294**	**0,127**	**0.132**	**0.865**	**2.597**	**0.160**	**2.765**	**0.024**	**0.028**	**0.208**
	**Dz**	A	0.686	0.940	0.173	0.044	0.197	1.394	1.876	0.106	2.565	0.031	0.022	0.0000
		B	1.369	1.550	0.299	0.148	0.086	0.247	1.375	0.123	**<LOD**	0.019	0.141	0.0575
		C	0.243	0.797	0.156	0.076	0.089	0.407	1.481	0.105	1.206	0.023	0.015	0.9674
	**Mean values**		**0.766**	**1.095**	**0.209**	**0.089**	**0.124**	**0.683**	**1.577**	**0.111**	**1.886**	**0.024**	**0.059**	**0.342**
	**mDz**	A	1.622	2.243	0.528	0.174	0.088	0.294	2.876	0.147	0.379	0.018	0.116	0.0000
		B	0.202	1.197	0.539	0.106	0.102	0.539	3.370	0.232	2.013	0.026	0.045	0.0377
		C	0.506	1.094	0.177	0.054	0.145	1.256	3.290	0.142	2.083	0.028	0.030	0.5942
	**Mean values**		**0.777**	**1.511**	**0.415**	**0.111**	**0.111**	**0.696**	**3.179**	**0.173**	**2.765**	**0.024**	**0.063**	**0.211**
	**H**	A	0.186	1.197	0.040	0.155	0.015	0.067	0.368	0.057	**<LOD**	0.005	0.008	0.0000
		B	0.554	1.580	0.264	0.130	0.313	1.519	2.362	0.189	3.273	0.053	0.019	0.0046
		C	0.184	1.555	0.163	0.263	0.128	0.857	0.930	0.156	2.945	0.008	0.012	0.3500
	**Mean values**		**0.308**	**1.444**	**0.156**	**0.183**	**0.152**	**0.815**	**1.220**	**0.134**	**3.109**	**0.022**	**0.013**	**0.118**
